# The Folate Concentration and/or Folic Acid Metabolites in Plasma as Factor for COVID-19 Infection

**DOI:** 10.3389/fphar.2020.01062

**Published:** 2020-07-16

**Authors:** Jesus Acosta-Elias, Ricardo Espinosa-Tanguma

**Affiliations:** ^1^ Facultad de Ciencias, Universidad Autónoma de San Luis Potosí, San Luis Potosí, Mexico; ^2^ Facultad de Medicina, Universidad Autónoma de San Luis Potosí, San Luis Potosí, Mexico

**Keywords:** SARS-CoV-2, folic acid, furin, viral infection, ace2

## Abstract

Pregnant women appear to be more susceptible to infectious diseases than women in reproductive age. According to the California Department of Public Health pregnant women were 9.6-folds more likely to be hospitalized during the 2009 influenza outbreak when compared to non-pregnant women in reproductive age. In contrast, it was reported that of 16,749 COVID-19 patients that were hospitalized in the UK, the probability for pregnant women to require in-patient care due to infection by SARS-CoV-2 was 0.95 versus non-pregnant women. Therefore 9.6/0.95 = 10.10, which brings us to the conclusion that pregnant women are 10.10-folds less likely to be hospitalized for a SARS-CoV-2 infection than for the 2009 H1N1 pandemic. Folic acid supplementation during pregnancy could be the factor that is protecting these patients against SARS-CoV-2 infection. Two independent papers that used informatic simulation proved that folic acid reduced the replication of this virus. One of them showed that folic acid inhibits the furin protease which the virus needs in order to enter its host cell, while the other one explained that folic acid inactivates protease 3CL*^pro^*, a protein that the virus needs to replicate. Nonetheless the probability that folic acid blocks two different proteins is very low, therefore the mechanism by which folic acid has apparently protected pregnant women during the COVID-19 pandemic has not been determined.

## Introduction

SARS-CoV-2 virus has caused a worldwide pandemic that is expanding, originating a serious public health crisis. Thus far a treatment has not been found and some countries have encountered a 7% death rate. Because of this high percentage, a vaccine is of the outmost importance but it is a year and a half away from being available for public use, this calls for the use of other medications that reduce the likelihood of presenting symptoms and are capable of slowing down the spread of the virus around the globe.

## Folic Acid

Folate is a hydro-soluble vitamin that classifies as part of the vitamin B complex. It is widely found in nature and a well-balanced diet provides the required daily needs for a healthy person. Many populations around the world have a diet that consists of crops such as potatoes, corn and rice on a daily basis, which are known to have a low folate concentration, originating a high deficiency prevalence (Gorelova et al., 2017). It is well known that folate intake is essential for human health because it prevents megaloblastic anemia and neural tube birth defects as well as cardiovascular disease, dementia, cognitive function alterations, osteoporosis and several types of cancer.

The food and Drug Administration (FDA) in the USA issued a regulation in 1996 requiring for all grain products to contain 140 mcg of folic acid (FA) per 100 g (Jacques et al., 1999), aiming to reduce the likelihood of new-born babies suffering from neural tube defects.

FA is a synthetic form of folate that is rarely found in nature. In order to process it into the organism after it is absorbed in the intestine (which is a very efficient process) it first needs to be reduced into dihydrofolate (DHF) and posteriorly into its active form tetrahydrofolate (THF) (Ducker and Rabinowitz, 2017).

Aside from diet, folate availability in humans is genetically modulated. There are several known polymorphisms of genes that codify for folate metabolizing enzymes, including 677C → T and the 1298A3C alleles of 5,10 methylene-THF reductase (MTHFR (MIM 607093)) among them (Yang et al., 2008).

The genetic alteration in 677C → T causes a thermolabile MTHFR enzyme with a diminished capacity to catalyze the conversion of 5,10-methylene-THF to 5-methyl-THF, a necessary step to obtain methionine from homocysteine. The presence of 677C → T is related to occlusive vascular disease (Amaral et al., 2017), neural tube defects (Zhang et al., 2019), Alzheimer’s disease (Rai, 2017) and hypertension (Shen et al., 2016).

Plasma and red blood cell (RBC) concentration also depends on the person’s ethnical group. For example, Non-Hispanic whites possess a 1,180 nmol/L RBC concentration in contrast to Non-Hispanic African Americans who have a 906 nmol/L (Pfeiffer et al., 2019). On the other hand, pregnant women who were taking 400 mcg of FA per day can reach a concentration of 2,862 nmol/L in RBC at delivery (Plumptre et al., 2015).

## Hospitalized Pregnant Women During 2009 A-H1N1 Pandemic

Pregnant women appear to be more susceptible to infectious diseases than women in reproductive age (Sappenfield et al., 2013). For example, during the 2009 influenza A-H1N1 pandemic the California Department of Public Health (CDPH) reported 94 pregnant women and 137 non-pregnant women who were in reproductive age that required in-patient care for Influenza H1N1 infection from April 23rd to August 11th, 2009 (Louie et al., 2010). The birth rate per 1,000 women in reproductive age in the USA during that year was 66.7 (Martin et al., 2011). Hence, for every 66.7 pregnant women there were 933.3 non-pregnant women in reproductive age. The probability that a pregnant woman will be hospitalized is obtained by means of the conditional probability equation: $P(A/E)=\frac{P(A\cap E)}{P(E)}$ where event A = hospitalization and event E = pregnant women. Substituting values we obtain *P*(*A*/*E*)=94/66.7 = 1.40929 (this result is not to be interpreted, it is merely a ratio). For the women in reproductive age and not pregnant $P(A/NE)=\frac{P(A\cap NE)}{P(NE)}$, where event A = hospitalization and event NE = non-pregnant women. Substituting values *P*(*A*/*NE*) = 137/933.3 = 0.14679. Therefore the probability for a pregnant woman to require being hospitalized when compared to a non-pregnant woman who is in reproductive age is 1.40929/0.14679 = 9.6. In other words, pregnant women were 9.6-folds more likely to be hospitalized for influenza H1N1 in 2009 than non-pregnant women in reproductive age.

## Hospitalized Pregnant Women During the Covid-19 Pandemic

According to the UK’s Office for National Statistics, the number women in reproductive age (15–45 years) by mid 2019 was 12,932,446 (Park, 2019) and the number of births in 2018 was 731,213 (Campbell, 2018). When this paper was written, there was no data regarding births in 2019. Extrapolating (731,213/(12,932,446–731,213)) * 1,000, the birth rate per 1,000 women in reproductive age was 59.929, thus it can be assumed that out of 59.929 pregnant women in the UK there are 940.071 non-pregnant women in reproductive age.

Docherty et al. (2020) reported that between February 6th 2PM and April 18th 2PM hospitals in England, Scotland and Wales admitted a total of 16,749 patients with COVID-19, 908 of those patients were non-pregnant women in reproductive age and 55 were pregnant women. The probability for a pregnant woman to be hospitalized is obtained by using again the conditional probability equation: 55/59.929 = 0.9177, whereas the probability for a non-pregnant woman to require in patient care is 908/940.071 = 0.9659. The probability for a pregnant women to be hospitalized when compared to a non-pregnant woman in reproductive age is 0.9177/0.9659 = 0.95.

In a study conducted at the New York Presbyterian Allen Hospital and Columbia University Irving Medical Center between March 22nd and April 4th 2020, analyzed 216 pregnant women who delivered infants and were tested for SARS-CoV-2 obtaining the following data: 84.6% were negative, 13.5% were asymptomatic and positive and only 1.9% were positive and symptomatic (Sutton et al., 2020).

## Hospitalized Pregnant Women During the Covid-19 Pandemic vs 2009 A-H1N1 Pandemic

Pregnant women are more susceptible to different infections, but in the case of SARS-CoV-2 they appear to be protected. If we compare with the 2009 H1N1 pandemic we can see that the magnitude of this protection is 9.6/0.95 = 10.10. Furthermore, during the H1N1 pandemic no ethnic differences were reported among pregnant patients who required being hospitalized (Louie et al., 2010).

Knight et al. (2010) reported that out of all pregnant women that were hospitalized in the UK between March 1st and April 14th 2020 due to an infection by SARS-CoV-2, 56% were black women or belonged to other ethnic minorities. The estimated incidence per 1,000 maternities of admission with SARS-CoV-2 infection was 28.4 for black women and 3.5 for white women. Therefore the black pregnant women are 8 folds (28.4/3.5) more likely to require in-patient care when compared to white pregnant women.

By June 13 of this year, Black Americans represent 12.4% of the population in the U.S., but they have suffered 24.3% of known COVID-19 deaths (Egbert et al., 2020). Using conditional probability equation, it can be estimated that Black Americans have a 2.27-folds more probability of dying from this infection than the rest of the ethnic groups in the U.S.

Thus, there is a possibility that FA supplementation in pregnant women confers them a protection that diminishes the likelihood of requiring hospitalization for SARS-CoV-2 infection by 10-fold when compared to the need for in-patient care during de 2009 H1N1 pandemic.

It may be that pregnant women who get infected abandoned their FA supplementation or did not take it as prescribed. Kinnunen et al. (2017) reported that during pregnancy, supplement use of FA was most common in European women (65.7%) and least common in Middle Eastern (29.4%) and African women (29.0%). There is also the possibility that some patients possess a polymorphism that alters their folate metabolism which places them at a higher risk for getting infected with SARS-CoV-2 as well as an increased probability of being symptomatic or requiring in-patient care.

## Discussion

On this paper we presented evidence that suggests that pregnant women possess a protective factor that decreases their chance of being hospitalized due to a SARS-CoV-2 infection by ten folds compared to the data obtained during the 2009 H1N1 pandemic. We propose their FA intake during pregnancy as the likely protective factor, however the means by which this is accomplished is still unclear. There are two independent works that have shown by computer simulation that FA can reduce the replication of this virus. In one of them (Sheybani et al., 2020) have shown that FA can inactivate the furin (Braun and Sauter, 2019) endoprotease. This endoprotease is crucial for the SARS-CoV-2 virus to enter its host cell (Coutard et al., 2020; Hua et al., 2020). While the other one (Serseg et al., 2020) explained that FA inactivates protease 3CL*^pro^*, which is vital in the replication of all coronaviruses (Min-Feng et al., 2005). The virus that originated the 2009 H1N1 pandemic did not need a furin endoprotease or a 3CL*^pro^* protease in order to replicate (Fujioka et al., 2018), however it seems very unlikely that FA could inhibit two different proteases. FA’s half-life is approximately 1.53 ± 0.46 h (Loew et al., 1987), although in the 10 h following its administration it could still be detected in small concentrations in plasma. It may be that SARS-CoV-2 virus inactivation is through THF molecules. With a 400 mcg FA supplementation per day pregnant women may reach concentrations as high as 2,323 nmol/L in RBC and 52 nmol/L in plasma at 12–16-week gestation (Plumptre et al., 2015). If this were the case, the severity of the infections may be inversely proportional to the concentration of certain folate molecules in the body. In other words, the mechanism by which FA supplementation protects pregnant women against SARS-CoV-2 is uncertain.

## Conclusions

Certain evidence seems to suggest that FA suplementation could be a protector against infection by SARS-CoV-2. Pregnant women seem to have a lower probability of acquiring this infection and those who are infected have a higher chance of being asymptomatic. On the contrary, pregnant women of ethnic groups who have a low folate RBC concentration secondary to genetic characteristics and/or does not receive FA supplementation have a higher hospitalization rate. This evidence shows that FA supplementation may protect against SARS-CoV-2.

## Author Contributions

All the authors have contributed equally to the development of this work.

## Funding

This work was partially supported by the Universidad Autónoma de San Luis Potosí.

## Conflict of Interest

The authors declare that the research was conducted in the absence of any commercial or financial relationships that could be construed as a potential conflict of interest.
